# The protective effect of walnut oil on lipopolysaccharide–induced acute intestinal injury in mice

**DOI:** 10.1002/fsn3.2035

**Published:** 2020-11-28

**Authors:** Fujun Miao, Chunlan Shan, Syed Aftab Hussain Shah, Rana Waseem Akhtar, Shuxiang Geng, Delu Ning, Xuanjun Wang

**Affiliations:** ^1^ College of Food Science and Technology Yunnan Agricultural University Kunming China; ^2^ Yunnan Academy of Forestry and Grassland Kunming China; ^3^ College of Animal Science and Technology Yunnan Agricultural University Kunming China; ^4^ Pakistan Scientific & Technological Information Center Quaid‐i‐Azam University Campus Islamabad Pakistan; ^5^ Department of Veterinary and Animal Sciences Muhammad Nawaz Shareef University of Agriculture Multan Pakistan

**Keywords:** acute intestinal injury, lipopolysaccharide, protective effect, TLR4/NF‐κB, walnut oil

## Abstract

Walnut oil (WO) is widely used in traditional medicine, and it has become a dietary supplement in many countries. We isolated walnut oil from *Juglans sigillata* and evaluated its protective effects on acute intestinal injury, and Toll‐like receptor 4 (TLR4)/nuclear factor‐κB (NF‐κB) signaling pathway in lipopolysaccharide (LPS)‐induced mice was studied. The results showed that the LPS + WO group significantly decreased serum tumor necrosis factor‐α (TNF‐α), interleukin‐6 (IL‐6), and IL‐1β levels and increased the jejunum superoxide dismutase (SOD) and glutathione peroxidase (GSH‐Px) levels compared with the LPS group. Walnut oil ameliorated the pathological morphology of the LPS‐induced acute jejunum injury and decreased jejunum cells apoptosis rate and TLR4/NF‐κB protein expression. Furthermore, the expression of the TLR4/NF‐κB pathway key gene mRNA significantly reduced after treatment with walnut oil. This study concludes that walnut oil can exert the protective effect on LPS‐induced acute intestinal injury in mice by inhibiting the TLR4/NF‐κB signaling pathway.

## Graphical abstract

Walnut oil ameliorated the pathological morphology of the LPS‐induced acute jejunum injury and decreased jejunum cells apoptosis rate and TLR4/NF‐κB protein expression. Walnut oil can exert the protective effect on LPS‐induced acute intestinal injury in mice by inhibiting the TLR4/NF‐κB signaling pathway.

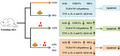

## 1 INTRODUCTION

The intestinal tract is the largest site of nutrient digestion, absorption, and the immune organ in the body. Intestinal mucosa, as the major mucosal layer in the body, plays an important role in maintaining intestinal homeostasis (Johansson & Hansson, 2016; Takahashi & Kiyono, 1999). However, under the influence of environment, diet, and other factors, the intestinal tract of the body is prone to trigger intestinal inflammation, leading to the occurrence of intestinal diseases (König et al., 2016). Inflammatory bowel disease (IBD) has become a global burden, whose pathogenesis is not fully understood and closely related to changes in intestinal oxidative stress, mucosal immunity, inflammatory factors, and gut microbiota (Ng et al., 2017). Toll‐like receptors (TLRs) are intermediaries among intestinal epithelial cells, microbes, and immune system and can participate in the development of IBD by regulating mucosal homeostasis, mucosal immunity, and gut microbiota (Fernandes et al., 2016). At present, the main clinical drugs used for the treatment of IBD include glucocorticoid, immunosuppressant, and 5‐aminosalicylic acid, etc., which are expensive, possess high toxic, and side effects and prone to drug dependence when taken for a longer duration (Danese et al., 2016). Due to the increasing incidence of IBD in recent years, which seriously affects human health, the extraction of safe, low toxicity, or side effect‐free functional components from natural products has become a current research hotspot (Ng & Ananthakrishnan, 2019).

Walnut is a traditional medicine and food. Walnut oil is extracted from it, containing most of the nutritional health and pharmacological effects (Arslan, 2010; Tsamouris et al., 2002). Walnut oil is rich in polyunsaturated fatty acids (linoleic acid, α‐linolenic acid, etc.), vitamin E, melatonin, phospholipids, squalene, flavonoids, and other fat‐soluble compounds containing antioxidant, anti‐inflammatory, cholesterol lowering, anticancer, brain health, and other effects (Griel & Kris‐Etherton, 2006; Hayes et al., 2016). Daily consumption of walnut oil can increase the activity of antioxidant enzymes such as superoxide dismutase (SOD), catalase (CAT), glutathione peroxidase (GSH‐Px) and reduce the oxidative stress of the body (Fan et al., 2004). Walnut oil can reduce the expression level of tumor necrosis factor alpha (TNF‐α) in peripheral blood mononuclear cells, thus improving the anti‐inflammatory ability (Jiménez‐Gómez et al., 2009). In addition, alpha‐linolenic acid supplementation can protect the integrity of the intestinal wall of IBD patients and generate anti‐inflammatory compounds by restoring bacterial balance (Ananthakrishnan et al., 2017).

Lipopolysaccharide (LPS) is one of the commonly used methods to establish acute intestinal injury in mice. The intraperitoneal injection of LPS can induce intestinal inflammation through the intestinal wall (Jia et al., 2018; Qin et al., 2014). However, it is unclear whether walnut oil has a protective effect against LPS‐induced acute intestinal injury in mice. Therefore, LPS was used to establish a model of acute intestinal injury in mice, and the protective effect of walnut oil on their intestinal injury was evaluated by detecting changes in intestinal lesions, antioxidant enzyme activity, inflammatory factors, and mRNA transcription level of TLR4/NF‐κB pathway in this study. It is expected that the results of this study will provide new directions for the prevention and treatment of intestinal diseases and lay down scientific basis for the further processing of products.

## 2 MATERIALS AND METHODS

### 2.1 Walnut oil preparation

Walnut oil was extracted from *Juglans sigillata* (Yunnan, China) using hydraulic press technology. The walnut oil was filtered through a rough filter mesh and stored in brown bottles at 4°C. The relevant physical and chemical indexes of walnut oil used in this research were refractive index n^20^D, 1.48; acid value, 0.50 mg/g; peroxide value, 1.70 mmol/kg; iodine value, 153.00 g/100 g; and saponification value, 200.10 mg/g. The unsaturated fatty acid contents of the walnut oil were 91.25% which further contained 16.66% oleic acid (OA, C18: 1), 64.19% linoleic acid (LA, C18: 2), and 10.40% α‐linolenic acid (α‐ALA, C18: 3), respectively.

### 2.2 Animal experiments

The 40 Kunming (KM) clean mice (male, 5 weeks old, 22 ± 2 g) were purchased from Kunming Medical University (Certification No. SCXK (Dian) K2015‐0002). All mice were provided feed and water ad libitum. The temperature (25 ± 2°C) and humidity (45 ± 2%) were kept constant in the laboratory. This study was approved by the Animal Protection and Utilization Committee of Yunnan Agricultural University, China. After a week of acclimation, the mice were randomly divided into four groups (10 mice/group), including control group (Con), lipopolysaccharide group (LPS), LPS + walnut oil group (LPS + WO), and walnut oil group (WO). The LPS + WO and WO groups were intragastrically administered 2.5 ml kg^−1^ day^−1^ walnut oil dose for 4 weeks, respectively. On day 29, LPS group and LPS + WO group caused acute intestinal injury with intraperitoneal injection of LPS at a dose of 3 mg/kg. LPS was dissolved in sterile saline. Sterile saline was injected intraperitoneally at a dose of 5 μl/g in Con and WO groups, respectively. Six hours after injection, blood and intestinal tissues were sampled from each treatment group.

### 2.3 Detection of serum inflammatory factors

Blood was collected from the eyeballs of mice in each treatment group, and serum was obtained by centrifugation at 2,000 *g* and stored at −20°C. According to the instructions of mouse tumor necrosis factor‐α (TNF‐α), interleukin‐6 (IL‐6), and IL‐1β ELISA test kit (Sangon Biotech Co., Ltd.), the contents of inflammatory factors in serum were detected.

### 2.4 Detection of levels of SOD, GSH‐Px, and MDA in jejunum

For this, 0.5 g of jejunum tissues was homogenized in nine parts of normal saline to make 10% tissue homogenate; the levels of superoxide dismutase (SOD), glutathione peroxidase (GSH‐Px), and malondialdehyde (MDA) in jejunum tissues were detected according to the instructions provided on the kits (Jiancheng Bioengineering Institute), and antioxidant activities were measured at 490 nm using an ELx800™ Absorbance Microplate Reader (BioTek Instruments).

### 2.5 Observation of jejunum histological structure

The 0.5 cm jejunum samples were removed from each group and embedded in paraffin. The paraffin blocks were sliced at 5 µm with a sledge microtome (Typ RM 2235, Leica) and stained with hematoxylin and eosin (HE) method. Histological observations were performed at 400× magnifications (Olympus CX43 microscope).

### 2.6 Detection of jejunum cell apoptosis with TUNEL staining

The paraffin slices of jejunum were prepared as described in Section 2.5. The TUNEL staining was performed to evaluate the cell apoptosis with cell death detection kit (Roche Applied Science) following the manufacturer's instructions. 4′,6‐Diamidino‐2‐phenylindole (DAPI) counterstained nuclei and glycerol mounts, and images were captured under a fluorescence microscope (Eclipse C1, Nikon).

### 2.7 Immunofluorescence of jejunum TNF‐α/NF‐κB double labeling

The paraffin slices of jejunum were prepared as described in Section 2.5. The slices were then incubated overnight at 4°C with anti‐TLR4 antibody (1:900, GB12186, Servicebio Co., Ltd.) and anti‐NF‐κB p65 (1:1,500, ab16502, Abcam Inc.). The mix 594‐goat anti‐mouse IgG (1:200, SA00013‐3, Proteintech) and 488‐labeled goat anti‐rabbit IgG (1:400, GB25301, Servicebio Co., Ltd.) incubated for 1 hr in a dark environment. DAPI counterstained nuclei and glycerol mounts, and images were captured under a fluorescence microscope (Eclipse C1, Nikon).

### 2.8 Detection of jejunum TLR4/NF‐κB gene expression with qRT‐PCR

The primer sequences for qRT‐PCR used are shown in Table 1, and these were synthesized by Beijing Tsingke^®^ Biological Technology Corp (China). RNA was extracted from jejunum tissue according to manufacturer's instructions and cDNA was obtained by reverse transcription (Tiangen Biotech Co., Ltd.). RNA concentrations were measured using the Nanodrop 2000 spectrophotometer (Thermo Scientific). Quantitative reverse transcription PCR (qRT‐PCR) was performed using a Rotor‐Gene 3000™ real‐time PCR cycler (Corbett/Qiagen) with QuantiNova SYBR Green PCR Master Mix (Qiagen, 208052). The qRT‐PCR conditions comprised of predenaturation (95°C, 30 s) and amplification [(95°C, 5 s; Tm, 20 s; 72°C, 30 s), 40 cycles]. The final mRNA expression fold change relative to the control was normalized to β‐actin.

**TABLE 1:** Specific primers for target genes

Gene	Sequence (5′–3′)	Tm (°C)
β‐Actin	F‐CCTGCGGCATTCACGAAACTAC	59.5
	R‐ACTCCTGCTTGCTGATCCACAATC	
TLR4	F‐AGGCAGCAGCTCGAATTGTATC	55
	R‐TTCCATCCAACAGGGCTTT	
NF‐κB	F‐GACGATCTGTTTCCCCTCAT	57.4
	R‐GCTTCTCTCCCCAGGAATAC	
IL‐1β	F‐AATGAAAGACGGCACACCCA	57.4
	R‐GGAAGACAGGCTTGTGCTCT	
TNF‐α	F‐GGCAGGTTCTGTCCCTTTCA	58
	R‐CTGTGCTCATGGTGTCTTTTCTG	

Abbreviations: IL‐1β, interleukin‐1β; NF‐κB, nuclear factor‐κB; TLR4, Toll‐like receptor 4; TNF‐α, tumor necrosis factor‐α.

### 2.9 Statistical analysis

All data were presented as the mean ± *SD* (*n* = 3), and significance of differences between groups was evaluated by ANOVA (SPSS, version 23.0), followed by the Duncan post hoc test. Differences were regarded as significant and extremely significant at *p* < .05 and *p* < .01, respectively.

## 3 RESULTS

### 3.1 Effect of walnut oil on serum inflammatory factors levels

First, we examined whether serum inflammatory factors of LPS‐induced acute intestinal injury were affected by walnut oil ingestion. As shown in Table 2, the serum levels of TNF‐α, IL‐6, and IL‐1β in the LPS group were significantly increased compared with the Con group (*p* < .01), indicating the success of the modeling. The serum TNF‐α, IL‐6, and IL‐1β levels of mice in the LPS + WO group were significantly reduced compared with the LPS group (*p* < .01 or *p* < .05). The levels of blood inflammatory factors in WO group were able to downregulate, but there was no significant difference with Con group (*p* > .05). Indirectly, walnut oil could inhibit the expression of serum inflammatory factors of LPS‐induced acute intestinal injury in mice.

**TABLE 2:** Effect of walnut oil on serum TNF‐α, IL‐6, and IL‐1β levels

Group	TNF‐α/(μg/L)	IL‐6/(pg/ml)	IL‐1β/(pg/ml)
Con	0.49 ± 0.02 Aa	12.36 ± 1.08 Aa	3.90 ± 0.16 Aa
LPS	1.29 ± 0.15 Bc	27.22 ± 2.31 Bc	6.92 ± 0.21 Cc
LPS + WO	1.05 ± 0.12 Bb	22.74 ± 2.14 Bb	6.25 ± 0.18 Bb
WO	0.48 ± 0.04 Aa	11.67 ± 1.40 Aa	3.93 ± 0.18 Aa

Note

Data are presented as averaged expression levels and standard deviation of three repeats. Different uppercase letters in the tail of the same column indicate significant difference (*p* < .01). Different lowercase letters in the tail mark indicated significant difference (*p* < .05). The same letter in the tail indicates no significant difference (*p* > .05).

Abbreviations: Con, control group; IL‐6, interleukin‐6; LPS group, lipopolysaccharide group; LPS + WO, LPS + walnut oil group; TNF‐α, tumor necrosis factor‐α; WO, walnut oil group.

### 3.2 Effect of walnut oil on the levels of SOD, GSH‐Px, and MDA in jejunum

The levels of SOD, GSH‐Px, and MDA are indicators of oxidative stress. As shown in Table 3, the SOD and GSH‐Px levels were significantly decreased (*p* < .01), while the MDA content was significantly increased in LPS group compared to Con group (*p* < .01), suggesting that LPS can significantly reduce the intestinal antioxidant level. In contrast, walnut oil administrations would increase the SOD and GSH‐Px levels (*p* < .05) and significantly decrease the MDA content (*p* < .01) compared to LPS group. It shows that walnut oil can improve the antioxidant capacity of LPS‐induced acute intestinal injury in mice.

**TABLE 3:** Effect of walnut oil on the SOD, GSH‐Px, and MDA in jejunum of mice

Group	SOD/(U/mg)	GSH‐Px/(U/mg)	MDA/(nmmol/mg)
Con	69.32 ± 2.32 Bc	48.63 ± 1.93 Bc	3.86 ± 0.14 Aa
LPS	52.65 ± 2.58 Aa	33.24 ± 1.42 Aa	4.82 ± 0.18 Bc
LPS + WO	57.97 ± 1.29 Ab	38.60 ± 2.74 Ab	4.33 ± 0.14 Ab
WO	71.64 ± 1.43 Bc	51.03 ± 1.21 Bc	3.90 ± 0.10 Aa

Note

Data are presented as averaged expression levels and standard deviation of three repeats. Different uppercase letters in the tail of the same column indicate significant difference (*p* < .01). Different lowercase letters in the tail mark indicate significant difference (*p* < .05). The same letter in the tail indicates no significant difference (*p* > .05).

Abbreviations: Con, control group; GSH‐Px, glutathione peroxidase; LPS group, lipopolysaccharide group; LPS + WO, LPS + walnut oil group; MDA, malondialdehyde; SOD, superoxide dismutase; WO, walnut oil group.

### 3.3 Effect of walnut oil on morphological changes in jejunum

**FIGURE 1:**
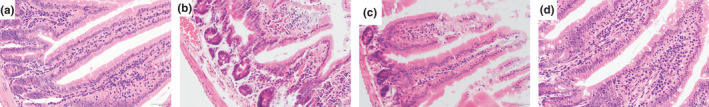
The morphological changes in jejunum of mice (HE staining, 400×). (a) Con, control group, (b) lipopolysaccharide group (LPS) group, (c) LPS + walnut oil group (LPS + WO), (d) walnut oil group (WO)

Considering the obvious effect of walnut oil on serum inflammatory factor levels and jejunum antioxidant capacity, we further investigated whether the histological changes of jejunum differed among the four groups with HE staining. As shown in Figure 1, the structure of jejunum in Con group and WO group was normal, and the villi structure was intact and arranged tightly without histological lesions. The jejunum injury showed that LPS injection resulted in atrophy of intestinal villi, shedding of epithelial cells, rupture of villi, and inflammatory cell infiltration, suggesting successful establishment of a mouse intestinal inflammation model. However, walnut oil treatment had shown a great protective effect, which had alleviated the pathological changes in the jejunum.

### 3.4 Effect of walnut oil on apoptosis and expression of TLR4/NF‐κB protein in jejunum

**FIGURE 2:**
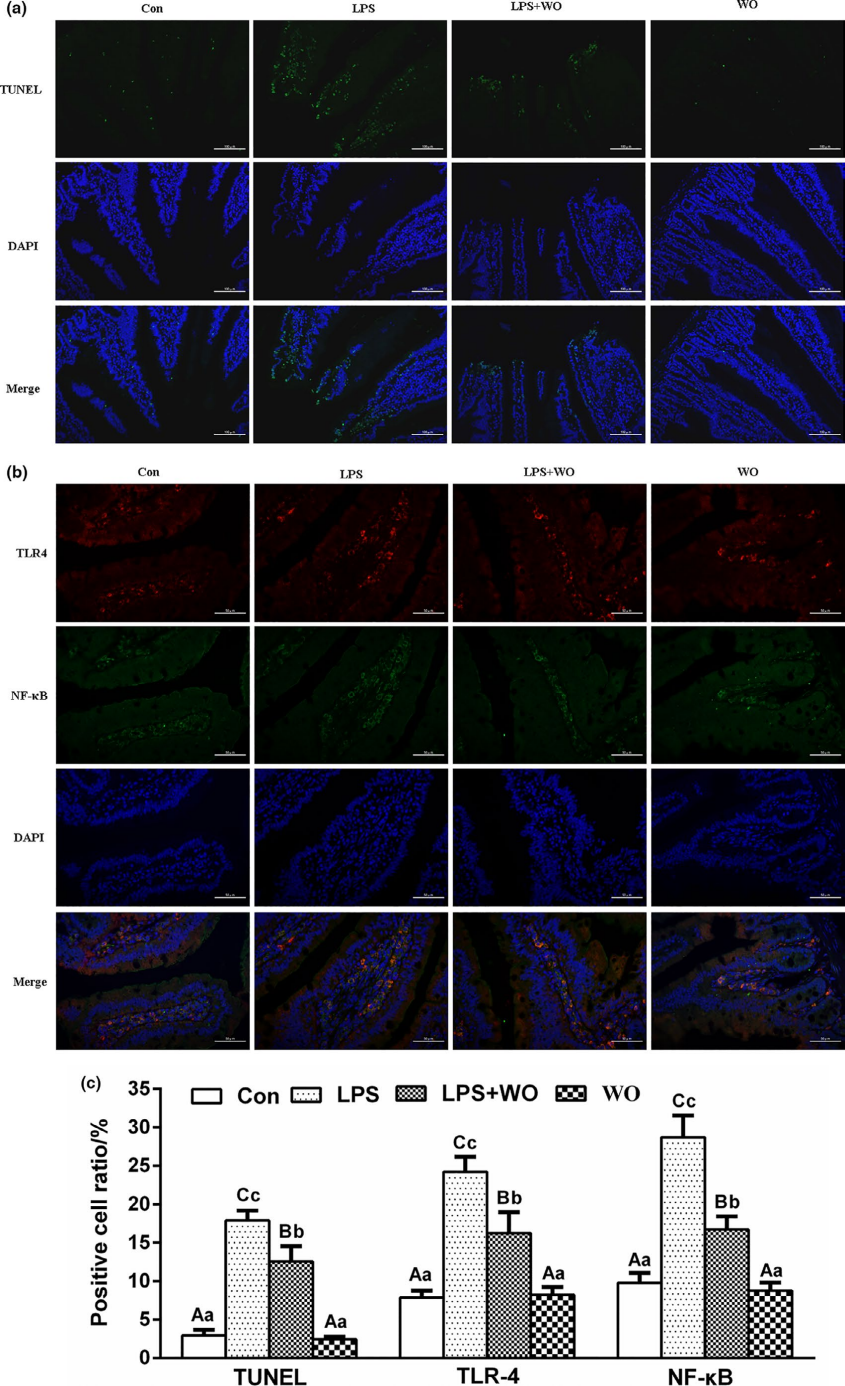
The TUNEL staining and TLR4/NF‐κB immunofluorescence double labeling staining in jejunum of mice. (a) TUNEL (200×), (b) TLR4/NF‐κB (400×), (c) apoptosis, TLR4, NF‐κB‐positive cell rate. Con, control group; LPS group, lipopolysaccharide group; LPS + WO, LPS + walnut oil group; WO, walnut oil group

Apoptosis is an inflammatory form of cell death and has been implicated in IBD. TUNEL staining results showed (Figure 2a) that apoptosis mostly occurred in the jejunum epithelial cells. Compared with the Con group, apoptosis‐positive cells in LPS group increased significantly (*p* < .01), (Figure 2c). Walnut oil treatment had significantly decreased the number of apoptosis‐positive cells (*p* < .01, vs. LPS group). There was no statistically significant difference in apoptosis rate between WO group and Con group (*p* > .05). It is suggested that walnut oil can prevent LPS‐induced apoptosis of jejunum epithelial cells.

Furthermore, immunofluorescence double labeling staining was used to detect the expression of TLR4 and NF‐κB protein in the jejunum of each treatment group. As shown in Figure 2b, TLR4‐positive cells were expressed in red; NF‐κB‐positive cells were expressed in green; and TLR4‐ and NF‐κB‐positive cells were mainly expressed in the lamina propria and epithelial layer, and most of them were coincided. Compared with the Con group, the rate of jejunum TLR4‐ and NF‐κB‐positive cells in the LPS group increased significantly (*p* < .01), (Figure 2c). However, the rates of jejunum TLR4‐ and NF‐κB‐positive cells in the LPS + WO group decreased significantly compared to LPS group (*p* < .01). There was no statistically significant difference in apoptosis rate between WO group and Con group (*p* > .05). It indicates that walnut oil could reduce the expression levels of TLR4 and NF‐κB in the injured intestine and reduce the occurrence of inflammatory response.

### 3.5 Effects of walnut oil on the mRNA expression of TLR4/NF‐κB pathway in jejunum

**FIGURE 3:**
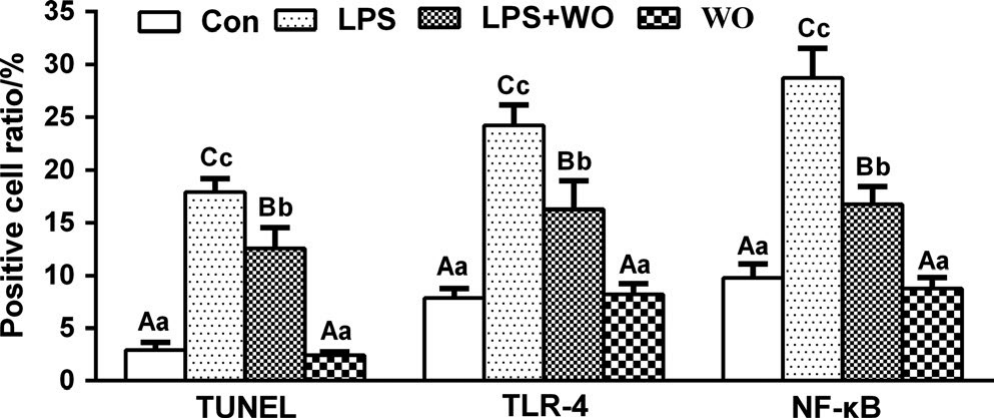
The relative mRNA expression of TLR4, NF‐κB, TNF‐α, and IL‐1β in jejunum of mice. Con, control group; LPS group, lipopolysaccharide group; LPS + WO, LPS + walnut oil group; WO, walnut oil group

We further attempted to determine the effect of walnut oil on TLR4/NF‐κB signaling pathway. As shown in Figure 3, transcription levels of TLR4/NF‐κB pathway key factors TLR4, NF‐κB, TNF‐α, and IL‐1β mRNA were significantly increased in LPS group compared to Con group (*p* < .01). Compared with the LPS group, the transcription levels of TLR4 and NF‐κB mRNA in the jejunum of LPS + WO group were significantly reduced (*p* < .01), and those levels of TNF‐α and IL‐1β were also reduced (*p* < .05).

## 4 DISCUSSION

Walnut is one of the largest nuts in the world and its oil has a good fatty acid structure (the ratio α‐ALA/LA is close to 1:4), giving it a variety of biologically active functions (Vinson & Cai, 2012). To the best of our knowledge, this was the first study to investigate the beneficial properties of walnut oil on the LPS‐induced acute intestinal injury in mice. LPS is a unique chemical component of the cell wall of Gram‐negative bacteria and composed of lipids and polysaccharides. After intraperitoneal injection into the body, it leads to increased permeability of the intestinal mucosa, triggers intestinal oxidative stress, and activates TLR4/NF‐κB inflammatory pathway, which in turn induces necrosis and apoptosis and promotes intestinal inflammation (Lu et al., 2008; Wang et al., 2020).

The antioxidant capacity of the body's defense system is closely related to inflammation. SOD and GSH‐Px are important antioxidant enzymes, and MDA is the end product of lipid peroxidation, which can reflect the body's antioxidant level (Li et al., 2014). Therefore, the activity of antioxidant enzymes in the intestinal tissue and the secretion levels of TNF‐α, IL‐6, and IL‐1β inflammatory factors in the serum can reflect the degree of intestinal inflammation and injury. In this study, the levels of serum TNF‐α, IL‐6, and IL‐1β inflammatory cytokines were significantly increased and reduced intestinal SOD and GSH‐Px antioxidant enzyme activities in LPS group. In contrast, walnut oil could significantly improve the antioxidant capacity and reduce the release of inflammatory factors of LPS‐induced intestinal injury. Previous studies found that consuming walnut oil not only increases the activity of antioxidant enzymes such as SOD and GSH‐Px but also reduce the expression levels of proinflammatory factors such as TNF‐α, thereby improving the anti‐inflammatory ability (Fan et al., 2004; Jiménez‐Gómez et al., 2009; Zhang et al., 2004; Zhao et al., 2018). Furthermore, the HE staining was used to study the improvement and protection of walnut oil on the pathological structure of acute intestinal inflammation from the perspective of pathological morphology. Walnut oil pretreatment attenuated LPS‐induced morphology damage of jejunum tissue in mice.

However, the precise pathologic mechanism during IBD is still controversial; increasing evidences from experiments and clinical patients indicates that unrestrained proinflammatory cytokine production is crucial to the pathogenesis of IBD. The TLR4/NF‐κB inflammatory pathway was identified as a critical mechanism of intestinal inflammation in the LPS‐induced injure model, and thus, negative regulation of TLR4/NF‐κB activity is essential for controlling inflammatory responses (Zhang et al., 2004). Indeed, these protective effects of walnut oil were mainly caused by suppressing LPS‐induced TLR4/NF‐κB pathway activation, less related genes (TLR4, NF‐κB, TNF‐α, and IL‐1β), and TLR4/NF‐κB proteins expression in our study. In addition, cell death and inflammation are fundamental characteristics in the initiation and development of IBD. The activation of TLR4/NF‐κB pathway promoted the occurrence of apoptosis (Lu et al., 2008). In current study, the jejunum epithelial cell apoptosis was determined with TUNEL staining. We found that LPS‐treated jejunum injury displayed characteristic features of apoptosis, and pretreatment with walnut oil desensitized jejunum epithelial cells to the inflammatory actions of LPS.

Although walnut oil has a positive role in intestinal anti‐inflammatory, its exact mechanism of action in intestinal inflammation still needs to be further explored, which may be closely related to repair and improvement of intestinal functional integrity and gut microbiota. Edible walnuts can increase the probiotic bacteria such as *Ruminococcaceae*, *Bifidobacteria*, *Clostridium*, etc., by regulating the structure and diversity of the gut microbiota of the body, and produce anti‐inflammatory factors short‐chain fatty acids (SCFAs) and interact with the host immune cells to maintain intestinal health (Bamberger et al., 2018; Holscher et al., 2018). In addition, walnut oil is safe and has been used as a health supplement or food additive, which indicates that it is a potential new treatment against IBD.

## 5 CONCLUSION

Walnut oil can alleviate LPS‐induced acute intestinal injury by increasing the activity of antioxidant enzymes and inhibiting the expression of related mRNA in the TLR4/NF‐κB inflammation signaling pathway. Walnut oil may be a potential treatment for IBD and has an excellent safety–benefit ratio.

## CONFLICT OF INTEREST

All authors declare that they have no conflict of interests.

## ETHICAL APPROVAL

All animals were kept in a pathogen‐free environment and provided feed and water ad libitum. This study was approved by the Institutional Animal Care and Use Committee of Yunnan Agricultural University, and all applicable institutional and governmental regulations concerning the ethical use of animals were followed.

## Data Availability

All data are fully available without restriction.
